# Exploring Cognitive Dissonance on a Ski Mountaineering Traverse: A Personal Narrative of an Expedition to ISHINCA (5530 m) in PERU

**DOI:** 10.3390/sports7120249

**Published:** 2019-12-11

**Authors:** Melissa Hart

**Affiliations:** Carnegie School of Education, Leeds Beckett University, Leeds LS6 3QS, UK; m.hart@leedsbeckett.ac.uk

**Keywords:** cognitive dissonance, strategies of dissonance reduction, characteristics of dissonance arousal and modes of reduction, consonant cognitions, attitude and behaviour change, autophenomenology

## Abstract

Through a personal narrative account, this paper explores the nature of the author’s cognitive dissonance experienced during a traverse of a high-altitude ski mountaineering objective (Nevado Ishinca 5530 m) in Peru’s Cordillera Blanca. The author experienced psychological discomfort in the ascent and a role of self in determining a continued commitment with the ski mountaineering challenge. Distraction, trivialization, act rationalization and finally attitude change were all used in attempt to reduce negative levels of cognitive dissonance. The lack of consonant cognitions to support abandoning the climb, the notion of free choice, the role of self-concept and self-esteem values motivated continued commitment until the negative levels of arousal subsided. Through a challenging mountaineering experience, I developed a greater self-awareness of the role of commitment to an objective which could be applied to other life events and experiences.

## 1. Introduction

High motivation, commitment and effort are required to achieve a mountaineering objective [1,2,3,4,5,6]. However, investigation into the process of continued engagement with, and maintenance of commitment to, a mountaineering objective is less widely investigated. In analysis of mountaineering autobiographies, the role of cognitive dissonance is considered in the construction of self and retrospective self-justifications for actions or decisions made whilst climbing mountains. Cognitive dissonance is experienced by a person when they have internal mental inconsistency of beliefs, values, goals or knowledge about the world [7]. As a result of the inconsistent nature of two or more pieces of related knowledge a person holds, individuals suffer from a form of mental discomfort or negative arousal. However, it is not clear how and why this is the case [7]. In order to rectify mental discomfort experienced as a result knowledge inconsistency, a person is motivated to make changes [7] to either justify a set of thoughts or actions or avoid situations that increase levels of discomfort. Broadly changes are therefore either attitudinal [8,9] or behavioural. In some previous studies the level of dissonance experienced is claimed to determine whether an attitude or behaviour change is likely [9]. However, it is difficult to consider mental and behavioural changes as separate or distinct as they are inextricably linked and form part of one another within a person. In addition, complex motivational and emotional traits influence decision making in the process of dissonance reduction suggesting it is not just a case of attitudinal or behavioural change [10].

Whilst most studies agree that the motivation for dissonance reduction is directly proportional to the level of discomfort experienced [11], ‘no research has unambiguously demonstrated the direction of this motivation—whether it is approach or avoidance orientated’ ([12], p. 1) Therefore, an action-based model of dissonance was proposed [13] ‘perceptions and cognitions can serve as action tendencies’ and decisions are made according to an approach-related process, so that thoughts are brought in line with goal-directed behaviours ([13], p. 36). However paradoxically, effective action can be interrupted by inconsistent cognitions, suggesting that sometimes thoughts cannot be brought into line with goal-directed behaviours [13]. In addition, the action-based model [14] is aligned to work on behavioural commitment [15], suggesting that people are approach-motivated to behave congruently (in actions and mindset) to a given commitment or goal and that this then reduces levels of dissonance. However, this does assume all people, in all contexts, would desire a reduction in dissonance.

**Figure 1:**
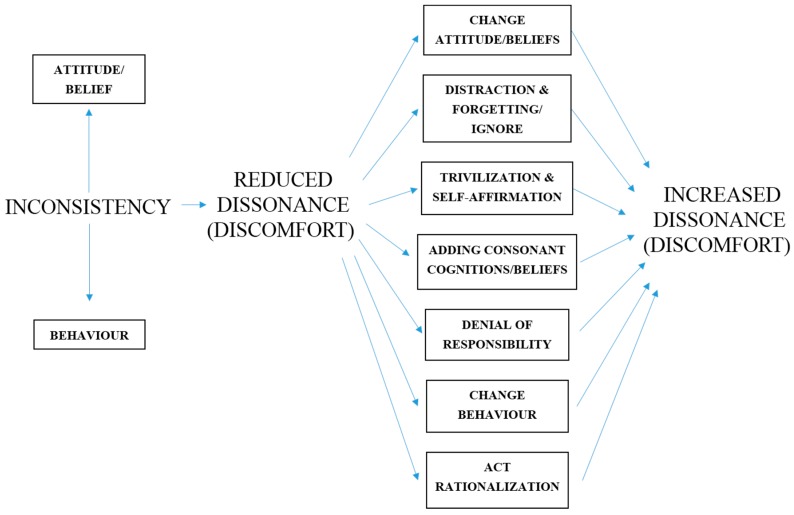
Dissonance reducing strategies.

The motivation to think or act differently may persist to reduce the levels discomfort associated with cognitive dissonance but the strategy of change is also important in understanding the process. There are several possible mental and behavioural approaches or strategies (in action or mindset) that could be used to reduce cognitive dissonance. Two reviews of other studies on dissonance [10,11] determine the types of strategies that may be adopted in cognitive dissonance reduction. These cognitive dissonance reducing strategies can be seen in the model in Figure 1.

Research in dissonance reducing strategies has focused on attitude change [8,9] and behaviour change [16,17], but neither considers the use of multi-strategies [11]. In addition, the simple strategies of distraction or forgetting [18] may be more efficient than attitude change [11], and trivializing the dissonant cognition though increasing salient values or self-affirmation [19] may reduce dissonant cognitions. However, if responsibility is needed for dissonance to develop [11], then a denial of responsibility [20] can be more effective than trivialization for reducing negative sensations. From a behaviour perspective, rationalizing inconsistent behaviour by adding consonant cognitions that support continued behaviour [21,22], or choosing action that is closer to the aspiring behaviour in a form of act rationalization [15], can reduce dissonance. However, the choice of strategy to reduce dissonance is difficult to predict [23], and there are limited studies into the state of emotional dissonance as opposed to the process of reduction and the role of attitude importance and trivialization [10,11,14,17,24]. Therefore, more contextually based study is required.

Based on other studies [11], some characteristics of dissonance arousal and the mode of reduction proposed may help to predict and analyse the process of dissonance reduction. Firstly, the magnitude of experienced dissonance is important in determining the nature of the mental or behavioural response, as well as secondly whether the dissonant inducting scenario it is freely chosen or requires compliance and finally the associated effort involved for completion [7,10,24]. In addition, the availability of a possible mode of dissonance reduction [11], and secondly the likelihood of success in achieving dissonance reduction [7], are factors affecting people’s choice of strategy. But none of the studies explain how individuals determine the effort required for dissonance reduction nor the chance of success. The effort required [7,9] can also limit possible change in attitude [8,9] or behaviour [16,17]. If the current behaviour is satisfying [7] then habits formed over a long period of time can be resistant to change [25]. ‘Studies have yet to compare how these paradigms potentially differ in (a) the level of dissonance magnitude aroused and (b) the selection of dissonance reduction modes’ [11:11], hence the need for further contextual studies. In addition, the impact of the affective emotional state of the individual [26] and whether it is a reoccurring level of discomfort [27] and the nature of the context [23] in which dissonance occurs, may influence the adoption of a mode of reduction [11].

## 2. Background

In 2018, following two winters living and skiing in the French Alps, I decided to travel to and complete a ski mountaineering expedition to the Cordillera Blanca, Peru with one climbing partner. The Cordillera Blanca is the most western part of the Peruvian Andes, north of the Peruvian Capital Lima. One of our ski mountaineering objectives was Nevado Ishinca (5530 m). Climbing Ishinca involves a day’s trek to its respective base camp. We used an Arriero (a person who transports goods using animals) and his two donkeys to carry our equipment to the basecamp. After at least a day of rest and a reconnaissance along the start of the route, an early start of 01:00 for Nevado Ishnica was required. A traverse of Ishinca was completed in 13 h. During the initial part of the ascent, I experienced periods of cognitive dissonance in starting and continuing with the climb. It is this initial commitment to the objective which is of interest as opposed to whether the summit was achieved. The strategies of dissonance reduction adopted along with the nature of dissonance arousal and modes of reduction are explored in this study.

## 3. Methodology

This broadly phenomenological [28] study uses autobiographical memory [29] to construct narratives (presented as vignettes) to explore a personal experience of cognitive dissonance and associated strategies of reduction in a specific ski mountaineering context. Not surprisingly, autobiographical memories have previously been used in analysis of high-altitude mountaineering studies or other outdoor activities to analyse actions and decision making, relationships with risk and other related psychological, social or environmental elements [1,5,6,30,31,32,33,34]. However, many of these examples involve analyses of others, as opposed to personal, experiences. Whilst readers may make some inherent comparison with other mountaineering stories, primarily I do not aim to situate personal experiences ethnographically within a culture of mountaineers. Instead these ‘recollections of specific, personal events’ ([35], p. 17) and the associated cognitive dissonance experienced are used by those that they pertain to in meaning making and construction of self [36], to understand acceptance of, and commitment to, experiences as they unfold. The context of a ski mountaineering experience stands as a metaphor for challenging life events and the successful completion of the objective is not important in the study. Hence, the research is autophenomenographical [37] in that it studies the phenomena of cognitive dissonance and associated strategies of reduction of the author as first participant rather than a cultural place or group, but with the added benefit of an understanding of cultural norms. It ‘is the study of the lived experience from the unique position of the individual that is engaged in the experience’ ([37], p. 3).

Two important phenomenological properties of autobiographical memory are ‘the sense of recollection and the belief that autobiographical memories are accurate’, both of which are heightened the more vivid the visual imagery in the remembered event ([38], pp. 79–80). There is high visual imagery in these autobiographical memories which also refer to specific places, times and events. However, they are also constructed within a discourse of other memories of similar events and life experiences, so memory boundaries and content are fluid. Whilst these autobiographical memories are long term, accessed from mental stores of information, they still involve significant assigned emotional and personal importance [35,39]. Emotions can change over time, through personal reflection [40], they can still exhibit stability over longer amounts of time [41] and this gives value for personal learning. In addition, as actor, agent and author [36], there are benefits of being able to see and feel the event as well as try to make sense of it that is not possible from the study of biographical accounts [42]. ‘Our stories are left incomplete if we omit the metaphoric and symbolic codes we use in narrating our subjective and personal realities’ ([43], p. 498). But this also means I am both a storyteller and a story analyst concurrently, as the narrative is constructed, the sequence and consequence offering an insight into my experiences [44] of cognitive dissonance in a specific ski mountaineering event. Informed consent was gained from my climbing partner to include their presence in my autobiographical memories. I used dissonance reduction strategies, characteristics of dissonance arousal and mode of reduction [11] as a framework for analysis.

## 4. Narrative Findings and Discussion

The negative psychological discomfort associated with cognitive dissonance [1], involves feelings of unease or tension [7,8,20]. However, there are few studies exploring the nature of dissonance arousal [1] or the characteristics of reduction [11]. In my ski mountaineering ascent of Ishinca (5530 m), I experienced a period of increasing cognitive dissonance before it abruptly dispersed. The greatest emotional discomfort was experienced in the first hour of the ascent after leaving the camp. However, prior to this the nature and magnitude of cognitive dissonance built as I prepared mentally and physically for the day ahead [7,10,24]:

I wake to hear other climbers passing the tent. It is about midnight. They are reasonably quiet, but my sleep is light and disturbed due to the knowledge that I must get up soon. My mind is already disturbed. Our alarm is set for 1am. I hate these early starts. I do not know if I hate them because it is early, and I must leave a warm sleeping bag to venture outside, or if I hate the uncertainty of what comes next. It is as if starting is the greatest problem.

‘Fear of the unknown’ is defined as ‘an individual’s propensity to experience fear caused by the perceived absence of information at any level of consciousness or point of processing’ ([45], p. 39). Intolerance of that uncertainty [46] can create cognitive, emotional and behavioural reaction, suggesting that cognitive dissonance could also be created by a *lack* of one set of information as opposed to inconsistency between sets of information [7]. However, the first hour of the climb had been completed the previous day, as a reconnaissance to a lunch spot, before returning to camp. Therefore, the first part of the ascent was ‘*known*’, even if the rest of the climb was not, further complicating analysis. A reaction to the cognitive dissonance might be to avoid the situation or ruminate [45], with a focus on both in this context. My attitude was focused around the uncertainty of what came next, the physical discomfort of lack of sleep, the context of an early start and venturing into the cold; attempting to accumulate cognitive reasons that would be consonant with not starting the climb [21,22,47]. Yet these consonant cognitions [21,22] were not salient enough in that moment to warrant abandoning the climb or even to voice concerns with my climbing partner.

My attitude was negative [10], which is a personal habit of recurring dissonance [25] in relation to mountain climbing objectives. Throughout all aspects of my life, I have regularly chosen to initiate new challenging objectives, but paradoxically I find committing to the actual start of some difficult to achieve. At the beginning of this ski mountaineering objective my level of cognitive dissonance was present but relatively low in magnitude [11], perhaps because I felt I was still able to make a choice about whether to commit [47]. I struggle with decision making in general, always wanting to keep options open or stop and think about it, and this manifests as greater cognitive dissonance in the mountaineering context given the requirement for immediacy of decisions. Within the simplicity of mountaineering context, the available choice of behaviour is often very limited—try to ascend or retreat, even if the nature of completing those actions is more complex. Paradoxically, therefore the limited choice of associated behaviour or available actions could create some greater levels of cognitive dissonance in my case, given my desire for lots of options. Yet the simplicity and adversity created by the mountaineering context does allow the opportunity to challenge self and transition through periods of cognitive dissonance which is often touted as a benefit for personal development and mental restoration [48]. Although, mental restoration had to wait as my cognitions were focused on mountain preparation in a somewhat anxious manner:

I want to sleep as I need the rest before the huge physical effort but it is limited in this situation as my mind is busy with checking and changing what has already been decided and considering every eventuality – have I packed the right equipment, what clothes will be best to wear, what will the ascent be like? I wonder how I was going to manage the ascent with ski boots on my feet and skis on my back. I think about the risk of falling somewhere on the climb, which has always been my main source of concern.

The dissonant condition is greater when real-life consequences exist to counter attitudinal behaviour [49]. In that sense the behaviour of carrying on with the climb is potentially counter attitudinal to a sense of survival and gives specific focus for further rumination [45] about risk levels, equipment and clothing. However, in the reconnaissance to a lunch spot the day before, I did not experience any environments in which I might fall, so this contextual thought could be trivialized [19]. I also had some confidence from having met others at the base camp and on the mountain—so it felt safer in terms of support or possible rescue. In order to consider whether the level of route was compatible with my ability there was opportunity to examine the appearance and discourse of others’ competence and compare it to my own [50] which proved favourable. So, whilst there were consonant cognitions [21,22] for not starting the climb, the magnitude of each was kept low and hence there was likelihood of successful motivating action [7]. I still had no salient nor valued consonant cognitions to provide me with reason to stop climbing, despite trying to find them, as further illuminated by the following narrative:

I slowly and methodically get dressed. Once outside the tent I discover it was not too cold. [The temperature did not get very cold till just before dawn when you were on the brink of wondering if you could cope with it and then the sun came up.] My climbing partner already had the stove going—hissing its way towards boiling water for tea and rehydrating breakfast cereals. I consider refusing to go—but I have no reason. I am angry with myself which makes matters worse. I drink and eat in thoughtful silence and busy myself by putting the final equipment in my rucksack. We are off.

My behaviour now mirrors my attitude to getting up and getting started in a slow procrastinating manner. I did not want to start the effort and was worried about potential risk I might encounter but none of these were salient enough justifications (consonant cognitions) for avoiding the climb [21,22] so any counter attitude was trivialized [19]. Deciding not to climb is the ‘easier’ physical choice, yet I do not make this easier choice, contrasting with the theory that the attitude [8,9] or behaviour adopted would be the one involving least effort to reduce dissonance [7]. Given my previous mountaineering experiences I had some tolerance to levels of cognitive dissonance [7] built through successful habits [25] of perseverance and resilience in these contexts. I knew I just needed to start. Therefore, I did not change behaviour [16,17] to match the attitude of not having to climb but aligned it to a more salient value, despite it giving me increased emotional discomfort [10]. The salient value for me is to persevere and the importance of this attitude [24] is great in the mountaineering context [2] but also secondly to reduce any negative emotional effects of *not* climbing.

I have implicit belief that whilst I do not want to start the climb, that any other behaviour would cause disappointment, regret and paradoxically resultant negative dissonance. Dissonance usually emerges when individuals perceive that they are free to engage in or refuse the counter-attitudinal behaviour and feel responsible for the negative consequences of their behaviour [10]. In this sense the decision to ascend is one of free will giving a counter attitudinal behaviour to the one requiring least effort. Having a high level of choice means attitudes are more likely to be consistent with behaviour [12]. Therefore, I avoided the potential negative dissonance by *not climbing* through taking responsibility for myself and my actions, indicating both a level of self-awareness and perhaps self-accountability in behaviour and attitudinal response [9]. In this sense my cognitions and behaviour are compatible, yet the mental tension remains which seems to oppose the traditional thinking on cognitive dissonance [7] or imply that there is greater complexity in the number and magnitude of inconsistent cognitions and behaviours occurring in this context. The nature of the number and salient value of these inconsistent cognitions (involving conflict with self) and behaviour increased the levels of cognitive discomfort as the following narrative illustrates:

The first hour was hard. I struggled to commit to and accept the objective, the action, the involvement. I knew it was about an hour to the place where we had had lunch the day before. This felt like a significant point in my mind, but I do not know why. I knew I had to just keep going to this place – literally one foot in front of the other. My head banged with the desire to stop, but not because I was finding it particularly hard work physically. I also did not really try to stop the thoughts but somewhat wallowed in the self-pity, and the perceived pain. I felt like an angry teenager, who could not explain or understand this sense of self. I was frustrated by my own thoughts and behaviour. But I could at least move my body forward at a consistent and even pace, although my head throbbed with negative black thoughts. I am not sure if my thoughts affected the nature of my pace, but I just wanted to get to that lunch spot.

It has been suggested that the magnitude of dissonance is related to the number of discrepant cognitions and the degree of importance of the cognitive elements [7,11,24]. Therefore, I was experiencing multiple inconsistent cognitions, they were of high importance, or both elements contributed to heightened negative arousal. This was our first ski mountaineering objective in Peru, which was less challenging in terms of height and difficulty of access and climb than other planned objectives. Yet I did not relish the easier challenge and perhaps put more cognitive pressure on myself to complete it successfully in order to boost self-affirmation [19]. In this sense the decision to ascend or retreat was further complicated by the resultant sense of self I would experience. It is therefore not just about committing to and accepting the objective but also about accepting self. As the level of emotional discomfort was so high, the magnitude of attitude change required was comparable [10] and so acted as a barrier to reducing the dissonance.

I attempted to strategically distract myself [18] and reduce the emotional discomfort associated with high levels of cognitive dissonance, through a form of ‘act rationalization’ [15]. I supported the conduct of physically moving, in order to create the most likely continued behaviour once past the lunch spot. I may have chosen *action* because similar actions have been successful in the past–a recurring habit which successfully reduced cognitive dissonance as a positive force, as opposed to a habit which is resistance to attitude change [25]. I find moving the body is something I can do when my mind is turbulent, especially if the action is simple and methodical. It is almost a release from the cognitive dissonance. It is meditative and calming. The repetition gives my body something to do, it occupies it, so I have space and time to think. I know I find swimming, running and walking all helpful in this manner. In addition, I acknowledge that the physical act was not as difficult as the mental act or attitude alignment. In that sense, the physical behaviour of starting the climb required the least effort so the counter attitudinal behaviour was not that negative after all [49]. Yet I experienced cognitive dissonance of extremely high magnitude at the time and theoretically responded by trying to reduce dissonance [10]. However, I experienced continued, if not increased dissonance, contrasting with dissonance theory [7], or showing a greater complexity to the process. It seemed that was sensed by my climbing partner:

Maybe my climbing partner sensed my emotional discomfort, as he asked if I was ok. I did not need or want his questions and that made matters worse! I could not stop the thoughts or put them to rest (although I did not particularly try any mindful techniques or meditative exercises). It made me irritated that he thought I was not ok, although I did not actually know if this was what he was thinking. His questions made me think he questioned my strength, my ability to manage and achieve the goal. Did I question these things about myself? I tried to explain the struggle to him and the need to just keep going for now.

Sometimes I consider mountaineering objectives to be the ambition of other climbers more than mine, perceiving a lack of free choice [47] and thereby inhibiting attitude alignment with behaviour. In addition, if I perceive a partners’ ease with the objective, as I struggle with my inconsistent cognitions, the greater my emotional discomfort, ‘your gain is my pain’ [50]. If I perceive I am subjected to some climbing partners’ assessment and critique, my emotional discomfort is further increased. And whilst I am struggling with my inconsistent cognitions, if I perceive their disappointment with me, in addition to my own, then my negative arousal escalates. Prosocial behaviour is correlated with comfort [49] and the increased social turmoil, even if it was just perceived by myself, contributed further to the magnitude of my cognitive dissonance, into feelings of anger, frustration and resentment.

However, anger and frustration can lead to increases in effort [26] which had a positive motivating effect in changing my attitude in this context [10]. Dissonance and motivation occur in the same brain zone and could explain the connection [12]. In this context, the cognitive dissonance contributed to an increased sense of stimulated alertness with a sense of associated increased energy which was paradoxically focused initially on further negative arousal rather than action. In addition, the heightened emotions and sense of arousal is a learned habit [25], making the goal physically and emotionally more rewarding if the cognitive dissonance is extreme and overcome. The high level of negative arousal in both personal and social aspects when combined with resilient action and the reaching of the previous lunch spot location led to a ‘bursting’ of tensions:

Once we were at the lunch stop from the day before the pain, anger and struggle strangely subsided, it disappeared suddenly. The dark crushing thoughts lifted spontaneously. I could now accept the challenge and started the ascent properly. Why did I have to go through this process of resistance of fighting with myself? What a waste of energy. Why was the first part the climb the hardest to achieve or was it passing this known line that I had to do to start for real? I had to keep going until I surrendered to the moment. I did not resist the task at this point, I accepted it.

Whilst self-concept or self-esteem may moderate dissonance processes, they may not cause dissonance [13]. Yet my sense of self was clearer, more motivated and self-esteem enhanced once I had arrived and passed the lunch spot, implying that whilst I had made the decision to physically move, I had not made the decision to emotionally commit to the climb until that point. ‘Once the individual decides on a course of action or makes a behavioural commitment, it enhances the value of the chosen course of action and reduces the value of the rejected course of action’ ([51], p. 36). Therefore, in contrast to some research [13] my attitude had to align to behaviour more than behaviour aligning to attitude, in order to create abrupt conflict resolution [52]. Viewing the course of action in a more positive manner will help the individual align action more effectively [51], which occurred here. The free choice paradigm in combination with act rationalization [15] processes emerged in a quick and almost automatic manner in this context [49]. However, I find it hard to explain why the lunch spot was key to the change in attitude and commitment to the climb, other than perhaps being the ‘true’ start to the unknown elements of the climb and any uncertainty induced fear [45]. The ski mountaineering objective did not start till we had moved beyond this location and it was where the negative arousal dispersed. Therefore, I would also argue that the nature of commitment and any associated action are fluid and not as simplistic as theoretical models [7,51] would suggest.

The original cognitive dissonance model [7] did not explain why cognitive discrepancy caused a negative emotive state [51] and the negative state I experienced was not easily explained by two opposing or contrasting cognitions. I experienced multiple inconsistent cognitions and attitudes to the knowledge, as well as behavioural actions at one time giving complexity to the cognitive dissonance. Like this context, individuals rarely just have just two inconsistent cognitions at one time, and it is more complex pattern than the original dissonance model [7] envisages. But what is interesting is the abrupt nature of attitude change I experienced and perhaps the energising force that was redirected from negative attitude to positive behaviour [10]. This implies the mountain environment can generate a context for life-affirming events that are congruent with personal change, resultant positive self-image and health.

Finally, the original cognitive dissonance theory [7] implies that behaviour and attitude are separate and can be changed independently from one another. Whilst my attitude to climbing did eventually become congruent with my behaviour and values, the notion that attitudes can be inferred from behaviour as consistent with self-perception theory [46] is too simplistic to explain my experiences in this context. Attitude is continually shifting according to context and so the importance of an attitude is only relevant at that one time and probably explains why the results of studying post dissonance attitudes have been contradictory [24]. Any attitude or behavioural change [16,17] as a result of reducing cognitive dissonance are dependent on the availability of new information (e.g., change in mountain weather, difficulty of the climb, or physical tiredness of the participant in this context) and the attitude salience [24]. Although post event analysis has enabled a greater self-awareness and understanding of my salient attitudes, the exact relationship between attitude and behaviour to reduce cognitive dissonance remains difficult to separate out and ascertain.

## 5. Conclusions

In the expedition to make a ski mountaineering traverse of Ishinca (5530 m) I experienced a range of cognitive dissonance reducing strategies: distraction [18], trivialization [19], act rationalization [15], and finally attitude change [8,9]. However, there was less behaviour change. My behaviour appeared more learned, stable and constant [38] than my attitude. Therefore, in contrast to some findings [13] attitudinal change aligned to behaviour. The lack of consonant cognitions [21,22] for abandoning the climb meant that negative cognitive arousal increased as the climb started. Yet even in the simplicity of the mountain environment, there was more than two inconsistent cognitions, so it is difficult to assess how attitudes and behaviour change as a result of one specific conflict. Attitudes are multidimensional and have affective, cognitive and behavioural dimensions [10].

In terms of the characteristics of dissonance, arousal, and modes of reduction, a variety of outcomes were experienced and explored. Firstly, the magnitude of dissonance arousal experienced during the ascent increased to a high point before abruptly disappearing altogether [49]. It is difficult to ascertain whether the negative arousal experienced was habitual, as a form of recurring dissonance, or to what extent the final attitude change played a part in the ultimate successes on the mountain. Could I have continued to climb with the same level of negative arousal? Affective state, through feelings of anger and frustration, was found to have a motivating role [26,49,52], although paradoxically the energy focused on negative arousal would have been more beneficial if it was directed at completing the climb itself. Reflection on previous experience (as a result of a recurring dissonance) allowed the author to use and build habitual behavioural strategies [25] which whilst offering some distraction paradoxically increased the levels of negative cognitive arousal experienced. My behaviour aligned to the salient values of tolerance and perseverance increasing the likelihood of a successful outcome. Thereby removing negative arousal associated with abandoning the climb and allowing self-affirmation [19]. However, less experienced mountaineers or individuals in similar dissonant contexts may not be able to draw upon reflective, stable and rational behaviour approaches [15] that operate in a contradictory manner to cognitions.

Whilst the notion of free choice in whether to climb had a role in attitude response, the contextual experiences alluded to a real complexity in how choice is perceived and acted upon. Attitudes, behaviours, social background, and values all affect how we see freedom of choice that is specific to contextual elements and the individual involved. So, it is difficult to ascertain the exact role of free choice in dissonance arousal or reduction in this case. However, the reduced decision-making choices or lack of available options that come from the simplicity experienced in a mountain context allows for individuals to reflect, examine and develop greater self-awareness to use in related experiences. Although in my case, the lack of available options might cause greater emotional discomfort. Paradoxically, the behaviour chosen was not the one requiring least effort, which further magnified cognitive dissonance. This suggests that neither attitude nor behaviour simply changes to reduce negative cognitive arousal.
